# *Sideritis scardica* Extracts Demonstrate Neuroprotective Activity against Aβ_25–35_ Toxicity

**DOI:** 10.3390/plants12081716

**Published:** 2023-04-20

**Authors:** Antonis Ververis, Kristia Ioannou, Sotiris Kyriakou, Niki Violaki, Mihalis I. Panayiotidis, Michael Plioukas, Kyproula Christodoulou

**Affiliations:** 1 Neurogenetics Department, The Cyprus Institute of Neurology and Genetics, Nicosia 2371, Cyprus; 2 Department of Cancer Genetics, Therapeutics & Ultrastructural Pathology, The Cyprus Institute of Neurology & Genetics, Nicosia 2371, Cyprus; 3 Department of Life and Health Sciences, School of Sciences and Engineering, University of Nicosia, Nicosia 2417, Cyprus

**Keywords:** *Sideritis scardica*, Alzheimer’s disease, plant extracts, neuroprotection, antioxidant, amyloid beta

## Abstract

Alzheimer’s disease (AD) is the most prevalent neurodegenerative condition, primarily affecting seniors. Despite the significant time and money spent over the past few decades, no therapy has been developed yet. In recent years, the research has focused on ameliorating the cytotoxic amyloid beta (Aβ) peptide aggregates and the increased elevated oxidative stress, two interconnected main AD hallmarks. Medicinal plants constitute a large pool for identifying bioactive compounds or mixtures with a therapeutic effect. *Sideritis scardica* (SS) has been previously characterized as neuroprotective toward AD. We investigated this ability of SS by generating eight distinct solvent fractions, which were chemically characterized and assessed for their antioxidant and neuroprotective potential. The majority of the fractions were rich in phenolics and flavonoids, and all except one showed significant antioxidant activity. Additionally, four SS extracts partly rescued the viability in Aβ_25–35_-treated SH-SY5Y human neuroblastoma cells, with the initial aqueous extract being the most potent and demonstrating similar activity in retinoic-acid-differentiated cells as well. These extracts were rich in neuroprotective substances, such as apigenin, myricetin-3-galactoside, and ellagic acid. Our findings indicate that specific SS mixtures can benefit the pharmaceutical industry to develop herbal drugs and functional food products that may alleviate AD.

## 1. Introduction

AD is the most prevalent form of dementia and an escalating neurological ailment that disproportionately affects older people. The disease’s primary symptoms are memory loss, confusion, and depression, which worsen over time until the patient is dependent on others to perform essential functions such as eating, moving, and speaking [1]. For the last 20 years, cholinesterase inhibitors and N-methyl D-aspartate receptor antagonists have been used to treat AD by providing palliative care only. Thus, a pressing need exists to develop a novel therapeutic approach to treat the disease more efficiently [2].

Even though the pathogenetic mechanism of AD is largely unknown, the generation of Aβ aggregates is considered one of the central initiating events of the disease, leading to neurodegeneration [3]. Hence, Aβ aggregate reduction is one of the main approaches currently being investigated for AD treatment, with some promising results already in clinical trials. These results led to the controversial accelerated approval by the United States Food and Drug Administration of a novel medication named Aducanumab, which targets amyloid beta peptide aggregates. However, the cost of this drug is high, and the European Medicines Agency has not approved it due to controversies regarding its effectiveness in improving clinical outcomes and safety. Therefore, there is still much work to be done to effectively treat AD [4,5].

Oxidative stress is another major AD hallmark, which leads to the generation of various toxic substances that can trigger disease development [6]. Previous studies have demonstrated that the consumption of antioxidants may lower the likelihood of AD onset and slow the disease’s progression [7,8,9]. Consequently, antioxidants can play a significant role in AD therapy [10]. Furthermore, oxidative stress and amyloid plaques may be interconnected in a way where the increased presence of oxidative stress is associated with the development of amyloid plaques, which in turn further elevate oxidative stress. The simultaneous restraint of these two phenomena may prove beneficial for AD patients [11]. Bioactive compounds or extracts from medicinal plants have important antiamyloid and antioxidant properties. Some of them may have the capacity to be used as future AD drugs, depending on their effectiveness and safety profile [12]. Thus, various medicinal plants can be useful in increasing the availability of ingredients or constituents for drug development, maximizing drug efficiency, and decreasing costs [13,14,15]. Presently, many medicinal plant species are considered sources of functional food with health-beneficial properties for AD [16].

There are more than 150 species in the genus *Sideritis*, which is a member of the Lamiaceae family, growing primarily in mountainous regions, with the majority located in the Mediterranean countries and Macaronesia. The genus name comes from the Greek word for iron, “sideros”, since these species were used in the ancient era to treat battle wounds caused by iron weapons. Its well-known health-beneficial properties led to its use in traditional medicine [17]. *Sideritis* taxa are mainly used to produce herbal teas to treat colds, flu, and bronchitis, and for the relief of mild gastrointestinal discomforts. A relevant herbal monograph (EMA/HMPC/39453/2015) on the traditional usage of four *Sideritis* species in herbal tea form has been compiled [18]. Recent research has shown *Sideritis* spp. to possess antioxidant, anxiolytic, anticholinesterase, anti-inflammatory, analgesic, antimicrobial, and antifungal properties [18,19,20,21,22]. Furthermore, a randomized, double-blinded, placebo-controlled clinical trial has shown that the daily intake of a *Sideritis euboea* extract enhances the growth of probiotics in the intestine [23].

*Sideritis scardica* (SS), with the alias “mountain tea”, is a medicinal, aromatic plant commonly used in Mediterranean countries to prepare medicinal herbal infusions. Mountain tea has a long tradition of usage in the Balkans ethnomedicine for treating the common cold and gastrointestinal disorders, while it is thought to maintain antioxidant and anti-inflammatory properties [24,25,26]. Extracts derived from SS have high contents of flavonoids and phenolics, substances with many pharmacological effects, such as antioxidant, anticancer, and antiviral properties [27,28,29]. Furthermore, methanolic and natural deep eutectic extracts of SS have demonstrated antiaging potential as well; ethanolic extracts showed remarkable anti-inflammatory, antimicrobial, and antioxidant capacities, while an SS dichloromethane extract exhibited anti-inflammatory activity [30,31,32,33]. SS hydroalcoholic extracts inhibit the reuptake of serotonin, noradrenaline, and dopamine. Therefore, they can potentially be used in treating neurotransmitter imbalances associated with various mental disorders [34]. Additionally, psychostimulant and antidepressive properties were exhibited in rats [35]. Regarding AD, SS extracts proved to reduce Aβ aggregation and toxicity in AD mouse models, in *Caenorhabditis elegans*, and in neuronal cell lines [36,37,38]. Especially in mice, they improved memory and learning and enhanced α-secretase expression, favoring the non-amyloidogenic processing of the amyloid precursor protein, while in a recent study, SS aqueous extracts reduced anxiety and memory loss [36,39]. In cell lines, SS extracts reduced tau hyperphosphorylation, another AD hallmark, and promoted the non-amyloidogenic pathway [38]. In other studies, SS extracts improved cognition in healthy individuals and patients with mild cognitive impairment (MCI), a condition regarded as a possible AD precursor [40,41,42]. To this end, a mixture of SS and *Bacopa monnieri* extracts positively impacted patients with MCI, in a double-blind, randomized, placebo-controlled clinical trial [43]. Finally, a similar parallel groups trial in healthy individuals exhibited that daily consumption of two mountain tea dosages boosted cerebral blood flow, reduced anxiety, and elevated visual sustained attention and working memory [44].

Here, we successively generated eight distinct SS extracts using solvents of different polarity levels and evaluated their antioxidant and neuroprotective capacities to assess their potential in developing functional foods targeting AD. The SS plants were obtained for experimental cultivation in Northern Greece. Various in vitro methodologies were employed to estimate the various SS extracts’ ability to function as neuroprotective agents.

## 2. Results

### 2.1. Chemical Characterization of the SS Extracts

The experimental pipeline was initiated by the content determination and quantification of the isolated extracts (petroleum ether (SSPE), dichloromethane (SSDM), methanolic (SSM), and initial aqueous (SSSW1)) and methanolic fractions (diethyl ether (SSDE), ethyl acetate (SSEA), butanol organic phase (SSB), and remaining aqueous (SSW2) isolated extracts) of *S. scardica*. To achieve this, ultra-performance liquid chromatography (UPLC) tandem mass spectrometry with electron spray ionization (ESI) in either positive or negative mode and spectrophotometric protocols have been utilized.

#### 2.1.1. High Levels of Phytochemicals Are Present in Several SS Extracts

Initially, we assessed the total contents of the main phytochemical categories such as phenolics (TPC), flavonoids (TFC), condensed tannins, and monoterpenoids in the SS extracts. This would help us to ensure the reproducibility of the results, to better characterize the extracts, and to obtain an initial insight into the antioxidant capacity levels of the fractions, as the above categories possess prominent antioxidant potential [45,46,47]. SSW1 was the extract with the highest TFC (~812 μmol catechin hydrate equivalents/gram, ~1024 μmol rutin equivalents/gram extract) and the highest TPC (~814 mg gallic acid equivalents/gram extract). Considerable TFC values were recorded for SSM, SSB, SSW2, and SSEA, while a relatively high TPC values were measured for SSM, SSDM, SSW2, and SSB. Conversely, the SSPE and SSDE fractions demonstrated low flavonoid and phenolic levels (Table 1 and Table 2).

The SSDM was the richest extract in the monoterpenoids (~25 μg of linalool equivalents per gram), while the SSW1 contained significantly higher concentrations of condensed tannins (~11 μg of linalool equivalents per gram), soluble proteins (~89 mg of BSA equivalents per gram), and soluble sugars (~17 mg of BSA equivalents per gram) in comparison with the rest of the fractions. In addition, SSW1 was significantly enriched with the pigments chlorophyll-a and -b.

#### 2.1.2. Identification of the Polyphenolics Present in the SS Extracts

**Figure 1:**
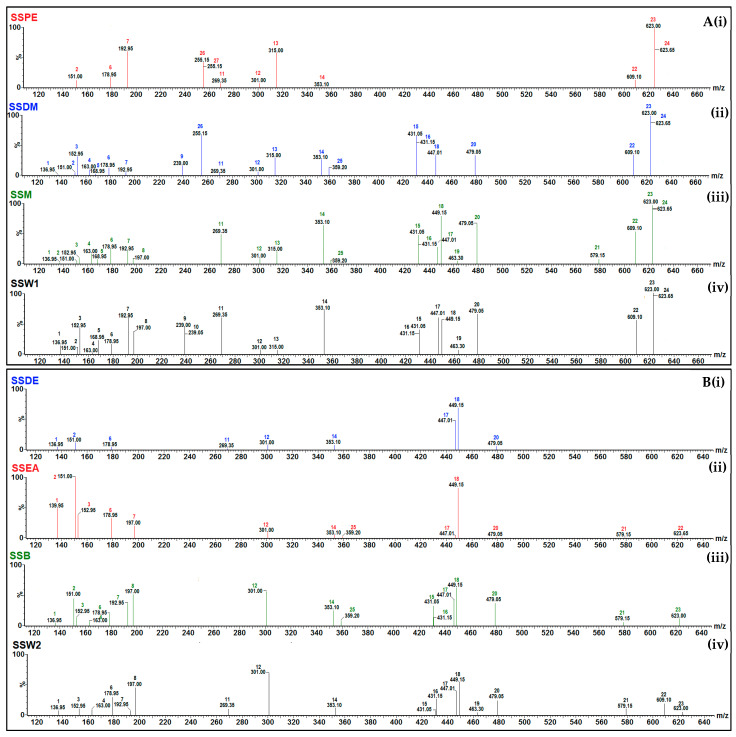
Selected ion recording (SIR) spectra: (**A**) (**i**) the petroleum (SSPE), (**ii**) dichloromethane (SSDM), (**iii**) methanolic (SSM), and (**iv**) initial aqueous (SSW1) isolated extracts and (**B**) the fractions of the methanolic extract (**i**) diethyl ether (SSDE), (**ii**) ethyl acetate, (**iii**) organic-phase butanol (SSB), and (**iv**) aqueous-phase butanol (SSW2) of *S. scardica*. Scanning (m/z; 120–650) was carried out utilizing collisions energies and m/z in the negative and positive electrospray ionization (ESI±) mode in accordance with the collision energy as presented in Table S1: **1**—4-hydroxybenzoic acid; **2**—vanillin; **3**—protocatechuic acid; **4**—p-coumaric acid; **5**—gallic acid; **6**—caffeic acid; **7**—ferulic acid; **8**—syringic acid; **9**—2′-hydroxyflavanone; **10**—7-hydroxyflavanone; **11**—apigenin; **12**—ellagic acid; **13**—isorhamnetin; **14**—chlorogenic acid; **15**—kaempferol-3-O-rhamnoside; **16**—apigenin-7-O-glucoside; **17**—quercetin-3-O-rhamnoside; **18**—luteolin-7-O-glucoside; **19**—quercetin-3-O-galactoside; **20**—myricetin-3-O-galactoside; **21**—naringin; **22**—quercetin-3-O-rutinoside; **23**—verbascoside; **24**—forsythoside; **25**—rosmarinic acid; **26**—4′-methoxyflavanone; **27**—5-methoxyflavanone.

For the identification of the commonest polyphenolic acids and flavonoids, selective ion recording (SIR) mode (Figure 1) was employed, whereas individual polyphenolic compounds were assessed through a multiple reaction monitor (MRM) transition with the use of commercially available external standards (Table S1). The International Conference of Harmonization’s standards were used to validate the analytical procedure (Table S2) [48].

Concerning all extracts, forsythoside A and verbascoside were the most enriched phenolic acids (39564 and 48,768 μg/g respectively) in SSW1, followed by chlorogenic acid (1987 μg/g) in the same extract. Myricetin-3-*O*-galactoside (~436 μg/g) and quercetin-3-O-rutinoside (~369 μg/g) were the most abundant flavonoids in SSW1 as well. SSW1 and SSM were the extracts with the higher concentrations in the identified compounds (Table 1 and Table 2).

### 2.2. SS Extracts Possess Important Antioxidant Properties

We used three approaches to investigate the antioxidant ability levels of the SS fractions. Firstly, we employed a DPPH assay, which measured the capacity of the SS extracts in scavenging the DPPH stable radical 1,1-diphenyl,2-picrylhydrazyl in terms of the extract concentration required to exhibit a 50% antiradical effect (EC_50_) [49]. This approach revealed a strong antiradical efficiency (AE) for the SSB extract, which surpassed the corresponding AE of the standard antioxidant Trolox, which was used as a positive control (AETrolox(DPPH⋅): 5.59). SSEA exhibited significant AE as well. The other extracts showed relatively weaker DPPH scavenging activity, especially SSDM and SSW2, whose radical scavenging activity values were close to zero (Table 3). We did not detect any scavenging ability in the SSPE.

Secondly, we utilized the FRAP (ferric-reducing activity power) assay, which measured the plant extracts’ potential to reduce Fe^3+^-tripyridyltriazine to Fe^2+^-tripyridyltriazine [49]. The results were similar to the DPPH assay, since SSB was the most potent antioxidant, followed by SSEA. SSM exhibited considerable potential, while the remaining mixtures were less effective, except for SSPE, which showed minor reducing ability (Table 3).

**Figure 2:**
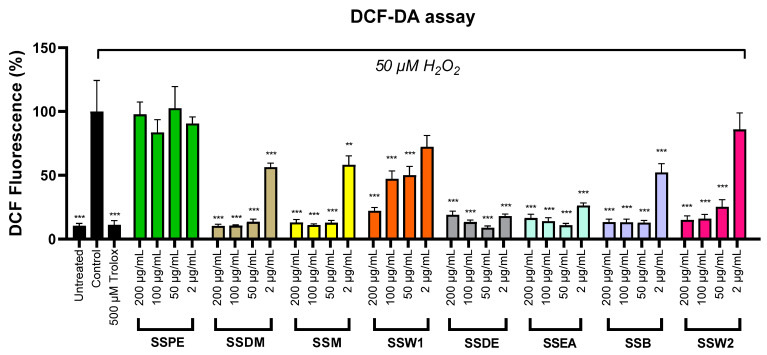
Effect of SS fractions on H_2_O_2_-induced oxidative stress in SH-SY5Y cells. The standard error of the mean (SEM) for five separate studies is displayed in the error bars. When compared to control cells incubated with 50 μM H_2_O_2_ alone, the symbols **, and *** indicate statistical importance at *p* < 0.01, and *p* < 0.001, respectively.

Finally, we assessed the antioxidant competence of the fractions in SH-SY5Y cells treated with hydrogen peroxide. We employed the DCF-DA assay, which measured the production of free radicals in cells [50]. The extracts were applied in a range of concentrations (2–200 µg/mL), and the results exhibited that they significantly lower the oxidative stress caused by hydrogen peroxide’s presence (Figure 2). This effect was more evident in concentrations ≥ 50 μg/mL. Below those levels, the protective action of the plant extracts started fading out. The only exception was SSPE, which did not show significant antioxidant activity. The EC_50_ values revealed strong antioxidant potential for SSEA, SSDE, SSB, SSM, and SSDM, while considerable activity was demonstrated for SSW2 and SSW1 (Table 3).

Overall, the SSB and SSEA mixtures showed substantial antioxidant potential in every assay. The DCF-DA assay also provided proof of a similar capacity for the other extracts, despite their relatively weaker antioxidant results in the DPPH and FRAP assays. The only exception was SSPE, which did not exhibit the antioxidant capacity in any of the employed approaches.

### 2.3. SS Mixtures Are Cytotoxic above a Specific Concentration

**Figure 3:**
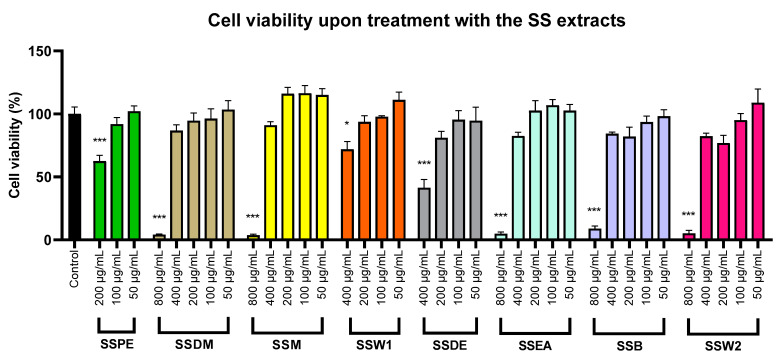
Determination of the maximum non-toxic concentrations of the different SS mixtures on SH-SY5Y cells. The error bars display the SEM from five separate tests. Note: * shows statistical significance of *p* < 0.05 and *** of *p* < 0.001 when compared to control untreated cells.

We investigated the possible cytotoxic effect of the SS mixtures in the SH-SY5Y neuroblastoma cells to define the maximum non-toxic concentration that cells could be incubated with in the neuroprotectivity assessment experiments. Each mixture was tested at a variety of concentrations, and it was shown that SSPE is cytotoxic at concentrations ≥ 200 μg/mL, SSW1 and SSDE at concentrations ≥ 400 μg/mL, while the remaining mixtures were characterized as cytotoxic ≥800 μg/mL (Figure 3). Table 4 shows the matching half-maximal effective concentrations (EC_50_), which were also computed.

### 2.4. SSDM, SSM, SSW1, and SSDE Exhibit Neuroprotectivity against Amyloid Beta Toxicity

**Figure 4:**
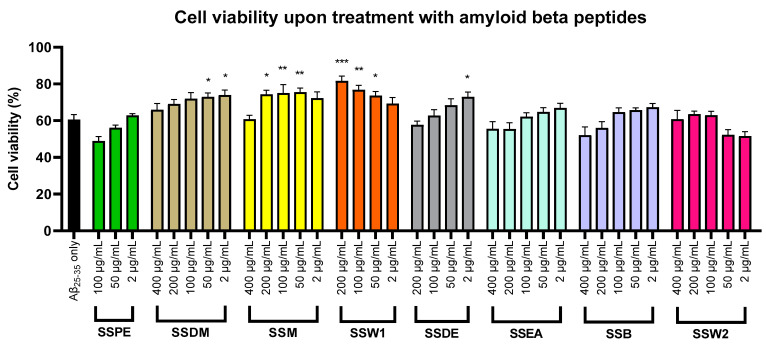
Determination of the neuroprotective capacity of the different SS mixtures against amyloid-beta-induced toxicity in SH-SY5Y. Error bars show the SEM values of five separate experiments. Note: * (*p* < 0.05), ** (*p* < 0.01), and *** (*p* < 0.001) when compared to cells treated solely with 30 μM Aβ_25–35_.

To investigate the possible neuroprotective potential of each mixture, SH-SY5Y cells were incubated with the highly neurotoxic amyloid beta 25–35 (Aβ_25–35_) peptides in the absence or presence of the SS extracts. Each SS mixture was tested in a range of concentrations determined through the cytotoxicity investigation (Figure 3). Four of the eight mixtures showed a statistically important capacity against Aβ_25–35_ neurotoxicity in specific concentrations. Specifically, SSW1 was the most potent and showed neuroprotectivity in concentrations of 50–200 μg/mL, SSM also at 50–200 μg/mL, SSDM at 2–50 μg/mL, and finally SSDE at 2 μg/mL only. The stronger antineurotoxicity was recorded after treatment with 200 μg/mL of SSW1, which increased the cell viability by 1.33-fold in comparison with cells treated solely with the neurotoxic amyloid peptides. The remaining four extracts (SSPE, SSEA, SSB, and SSW2) did not demonstrate a neuroprotective effect (Figure 4).

**Figure 5:**
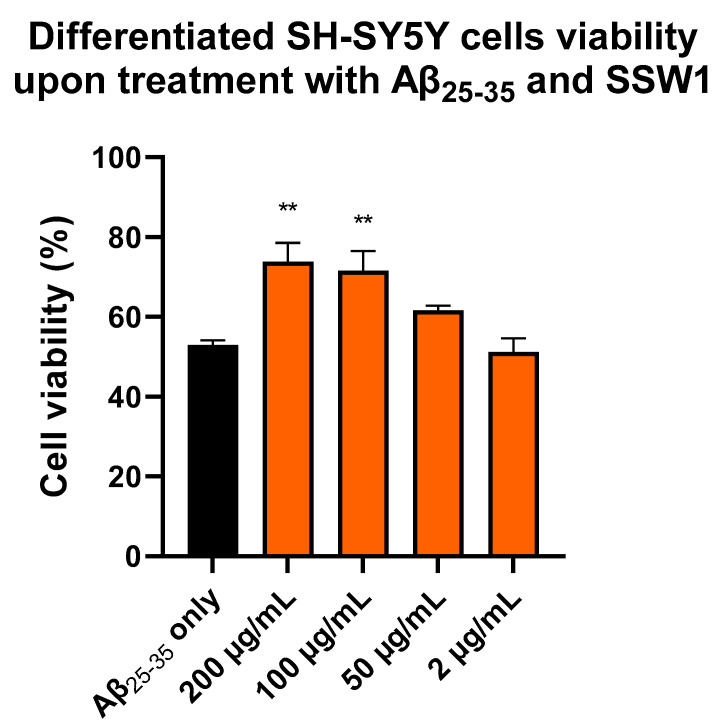
Determination of the neuroprotective capacity of various concentrations of SSW1 against amyloid-beta-induced toxicity in retinoic-acid-differentiated SH-SY5Y. Error bars show the SEM values of five independent experiments. Note: ** refers to *p* < 0.01 statistical significance compared with cells treated with 30 μM Aβ_25–35_ only (first column).

To confirm this finding, we tested whether SSW1, which showed the highest neuroprotective potential, could have the same effect in retinoic-acid-differentiated SH-SY5Y cells. Indeed, SSW1 showed a strong neuroprotective action against Aβ_25–35_ by partly restoring the viability of the cells treated with the neurotoxic peptides (Figure 5). In particular, while the Aβ_25–35_ treatment caused the viability of the cells to fall to ~53% compared with the untreated cells, the addition of 200 and 100 μg/mL SSW1 restored the cell viability to ~74% and ~72%, respectively. Treating cells with 50 μg/mL of SSW1 restored the cell viability to ~62% but was not statistically significant, while 2 μg/mL of SSW1 did not have any effect.

## 3. Discussion

Here, we present evidence that SS extracts possess neuroprotectivity against neurotoxicity caused by amyloid beta peptides and significant antioxidant potential. The methodology selected for the estimation of such potential has been successfully employed in previous studies investigating similar modes of action by other medicinal plant extracts [51,52]. The extraction process for the generation of the fractions favors the separation of the plant’s bioactive compounds at higher concentrations in the extracts [53]. Various solvents of different polarities were used to generate eight SS fractions of distinct secondary metabolites’ contents, as evidenced by the chemical characterization data. Thus, plant material fractioning enables a thorough examination of the plant’s relevant properties by maximizing the possibility of uncovering a health-beneficial potential that otherwise may remain inconspicuous.

In general, the mode of action of the various antioxidants can differ, so it is highly recommended to employ at least two different approaches when investigating the antioxidant activity of the heterogenous plant extracts [54]. Here, the antioxidant potentials of the fractions were assessed using three different techniques: DPPH, FRAP, and DCF-DA assays. These tests were used to determine the extracts’ capacity to scavenge free radicals, their reducing activity, and their antioxidant potential in living cells, respectively [49]. Regarding the neuroprotection experiments, Aβ_25–35_ peptides are the shorter Aβ forms that retain the cytotoxicity of the full-length forms (Aβ_1–42_) in a similar pattern; thus, the region they cover is considered the active region of the neurotoxic Aβ peptides [55,56]. Therefore, Aβ_25–35_ is a convenient tool that has been used in conjunction with the SH-SY5Y human neuroblastoma cells to establish a cell line model for AD [51,52,57,58].

For this work, the plant material originated from SS plants cultivated according to good agricultural practice, which assured a stable and high-quality plant material enriched with the desired components. In addition, cultivation ensured the reproducibility of this work’s findings because of the standardized plant material that was used, in opposition to plants originating from the wild, which are more susceptible to chemo-variations [59]. At the same time, a significant genetic adaptive variation has been shown between diverse natural SS populations [60]. Additionally, SS plants of different origins show remarkable differences in their phenolic and flavonoid contents, depending on genetic factors and the different geoclimatic conditions occurring in their habitats [54,61].

As demonstrated earlier in plants growing in Greece, Serbia, Bulgaria, and North Macedonia, the TFC and TPC testing showed the presence of significant levels of flavonoids and polyphenols in SS. The antioxidant potential of SS fractions, as evaluated by DPPH and FRAP assays, was shown to correlate positively with the presence of phenolics and flavonoids observed in previous studies [27,62]. To this end, SSB and SSEA had relatively high TFC values together with considerable TPC values and showed the most substantial antioxidant potential, in accordance with previous studies in wild SS plants and cultivars from Bulgaria showing similar results for SS extracts generated with the use of butanol and ethyl acetate [62,63]. The DPPH and FRAP assays showed a weaker antioxidant capacity for the remaining extracts, even though some of them demonstrated high TFC and TPC values, such as SSM and SSW1. These two extracts possess a considerable soluble protein content, and it has been documented that the interactions of phenolics with proteins can limit their reactive groups and antioxidant potential [64,65]. Furthermore, previous studies suggest that the antioxidant activity is not always correlated with the TPC and that it is dependent on the structure–activity relationships of the bioactive compounds [66,67,68,69]. Nevertheless, except for SSPE, the DCF-DA assay demonstrated that all SS mixtures exert important antioxidant activity in vitro. This potential can be explained by the existence of bioactive substances with documented antioxidant effects such as chlorophyll-a and -b, vanillic acid, 4-hydroxybenzoic acid, syringic acid, p-coumaric acid, caffeic acid, ferulic acid, verbascoside, and quercetin-3-*O*-rhamnoside, among others [70,71,72,73,74,75,76,77,78]. Notably, SSPE was the only extract that did not display any discernible antioxidant ability with any of the utilized approaches. This indicates that the non-polar solvent petroleum ether did not extract strong antioxidant compounds, contrary to the other solvents used here that possess higher polarity indexes. Similar findings were observed in other plants such as *Achillea*, *Phaseolus*, and *Mentha* species, and it is generally considered that polar extracts possess stronger antioxidant potential because of the polar solvents’ ability to more effectively isolate antioxidant compounds such as highly hydroxylated forms of phenols [79,80,81].

The higher extract concentrations exerted cytotoxicity on the cells, probably due to the presence of specific substances becoming cytotoxic above a specific concentration. Previously, SS extracts have been shown to cause cytotoxicity in rat glioma cells through oxidative stress induction and cell cycle arrest, leading to apoptosis and autophagy. Phenolics present in the SS extracts, such as apigenin and luteolin, cause similar effects and are considered at least partly responsible for the observed toxicity [63,82].

Four of the eight studied SS mixtures demonstrated a statistically important neuroprotective capacity against the cytotoxicity caused by amyloid beta peptides, further confirming the potential of SS to provide mixtures or constituents that can be beneficial to patients with AD. Previous studies have shown that methanolic, ethanolic, and aqueous SS extracts, as well as ethanolic partitions, can reduce Aβ_1–42_ aggregation and stop neuronal loss in cell lines and mouse and worm models of AD [36,37,38]. Specifically, in mice, SS extracts have been shown to decrease soluble Aβ_1–42_ and Aβ aggregation by enhancing the phagocytic microglia response of Aβ and inducing the expression of a-secretase ADAM10, which cleaves Aβ [36]. Here, we show that four SS extracts (SSDM, SSM, SSW1, and SSDE) can also reduce the cytotoxic activity of Aβ_25–35_, with SSW1 showing this potential in differentiated cells as well. In addition, for the first time, we present neuroprotectivity in SS extracts generated by using less-polar solvents, such as dichloromethane. Nevertheless, the more polar extracts, such as the methanolic and aqueous extracts, are the strongest in terms of neuroprotectivity, as also indicated by previous findings [36,37,38,39]. The presence of known neuroprotective SS compounds (Table 1 and Table 2), such as luteolin, apigenin, ferulic acid, chlorogenic acid, caffeic acid, ellagic acid, myricetin, quercetin, protocatechuic acid, gallic acid, vanillic acid, syringic acid, p-coumaric acid, kaempferol, verbascoside, and forsythoside A, likely confers Aβ_25–35_ anti-neurotoxicity to the four neuroprotective fractions presented here [83,84,85,86,87,88,89,90,91,92,93,94,95,96,97,98]. Notably, ellagic acid and myricetin-3-*O*-galactoside are enriched in these four fractions, proportionally to their neuroprotective potential.

Conclusively, SS extracts are characterized by antioxidant capacity and neuroprotectivity, which are properties that can target important AD hallmarks. Especially, the dichloromethane (SSDM), methanolic (SSM), initial aqueous (SSW1), and diethyl ether (SSDE) mixtures possess both desired qualities. At the same time, the butanolic (SSB) and ethyl acetate (SSEA) fractions demonstrate a striking antioxidant effect. Future directions should include the administration of the extracts—and especially of SSW1, which shows the highest neuroprotective potential—in AD mouse models to gather additional evidence regarding their potential against AD. Nonetheless, the results of this work strengthen the notion that SS can be exploited as an additional, affordable source of bioactive compounds or mixtures to be used in the development of herbal drugs, functional foods, and supplements, targeting AD.

## 4. Materials and Methods

### 4.1. Chemicals

Solvents: Water, acetonitrile, acetone, chloroform, and methanol, were obtained from Honeywell (Charlotte, NC, USA). LC-MS-grade formic acid was supplied by Thermofisher (Waltham, MA, USA). Sulfuric acid, phenol, hydrochloric acid, *n*-butanol, ethyl acetate, diethyl ether, petroleum ether, and dichloromethane were supplied by Sigma Aldrich (Saint Louis, MO, USA). DMSO was supplied by Santa Cruz (Dallas, TX, USA).

Analytical standards: All standards were supplied by Extrasynthese (Lyon, France) except catechin, rutin, ascorbic acid, mannose, and linalool, which were supplied by Sigma Aldrich, as well as apigenin, forsythoside A, and verbascoside, which were provided by Adooq Bioscience (Irvine, AB, Canada). Bovine serum albumin (BSA) was purchased from Thermofisher. Trolox, an antioxidant vitamin E derivative, was obtained from Abcam (Cambridge, UK).

Reagents: Aluminum chloride, sodium nitrite, sodium hydroxide, TPTZ, iron (III) chloride solution, ammonium iron (II) sulphate, Folin–Ciocalteu’s phenol reagent, thiazolyl blue tetrazolium bromide, 2′,7′-dichlorofluorescin, and DPPH were purchased from Sigma Aldrich. The amyloid beta peptides (Aβ_25–35_) were supplied by Genscript (Piscataway, NJ, USA).

Assay kits: A bicinchoninic acid (BCA) protein assay kit was supplied by Thermofisher.

Cell Culture: The cell culture reagents and media were supplied by Biosera (Nuaille, France).

### 4.2. Plant Material

The plant material was gathered in an experimental field at the Institute of Plant Breeding and Genetic Resources of the Hellenic Agricultural Organization “DIMITRA” in Thermi, Greece, from a population of *S. scardica* that had been cultivated under “organic” farming conditions. The germplasm of the initial population originated from the East Macedonian mountain area (Greece). The plant material was recognized by a plant taxonomist. The aerial parts of the three-year-old plants were gathered during flowering (June 2017) and air-dried under shadows, and the preparation of the extracts and other fractions was conducted at the Laboratory of Pharmacognosy, University of Nicosia, Nicosia, Cyprus. A voucher specimen was submitted to the same lab for future use, under the code number 0617-SdrtscELGO.

### 4.3. Preparation of SS Extracts

**Figure 6:**
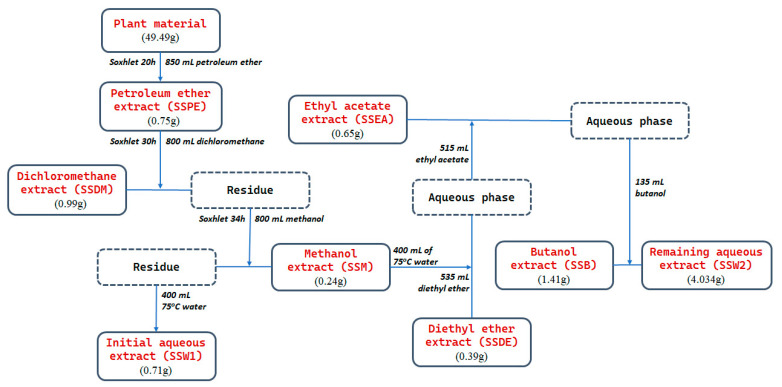
Graphical representation of the plant extraction process leading to the generation of eight distinct SS extracts.

The plant material (fixed-weight 49.49 g) was exhaustively extracted into a Soxhlet extractor with the solvents petroleum ether (850 mL), dichloromethane (800 mL), and methanol (800 mL) consecutively (for 20 h, 30 h, and 34 h, respectively), and the three collected fractions (SSPE, SSDM, SSM) were concentrated under vacuum to the point of dryness. The aerial parts of *Sideritis scardica* were then extracted with 400 mL of 75 °C water and the fraction (SSW1) was evaporated to dryness under reduced pressure. The dry remainder of the methanolic fraction was dissolved in 400 mL of 75 °C water, filtrated, and partitioned using the following solvents: diethyl ether, ethyl acetate, and n-butanol (20-fold of 26.75 mL, 20-fold of 25.75 mL, and 9-fold of 15 mL, respectively). The organic layers of these solvents (SSDE, SSEA, and SSB respectively) and the remaining aqueous extract (SSW2) were evaporated to dryness. This procedure is graphically presented in Figure 6. Before use, the extracts were dissolved in DMSO or methanol (1 mg/mL), passed through a 0.22 μm filter (cellulose nitrate; Sartorium Stedim, Guttenberg, Germany), and maintained at −20 °C in the dark.

### 4.4. Total Phenolic Content (TPC) Assessment

The TPC values of the fractions were evaluated with the use of a polyphenolic quantification assay kit (Bioquochem, Asturias, Spain) as per the kit directions. A gallic acid calibration curve (linear range: 0–0.8 mg/mL, y = 9.7x + 0.0155, R^2^ > 0.9962) was the basis for how the TPC values were computed. The analysis was carried out in triplicate, and the findings were presented as μg of gallic acid equivalents per gram of dry extract with standard deviations.

### 4.5. Total Flavonoid Content (TFC) Determination

With a few adjustments, the TFC measurements were carried out as previously published [99]. Briefly, 20 μL of aluminum trichloride (10% aqueous solution) and 20 μL of sodium acetate were added to 40 μL of every extract after it had been diluted with 120 μL of methanol (0.5 M aqueous solution). The resultant mixtures were left at room temperature for 40 min protected from light before having their absorbance quantified at 415 nm with a microplate reader (LT4500, Labtech, Heathfield, UK). The rutin (linear range: 0–2 mM, y = 0.6339x + 0.0144, R^2^ > 0.9997) and catechin calibration curves (linear range: 0–1.2 mM, y = 1.0711x − 0.0009, R^2^ > 0.9974) were used to calculate the TFC. The TFC was measured in μmol of catechin hydrate or rutin/g of dry fraction, and the measurements were performed in triplicate. The findings are presented as averages with SDs.

### 4.6. Total Condensed Tannin Content Assessment

The amount of condensed tannins was estimated using an earlier reported technique [100]. In a nutshell, one part of each extract was diluted with one part of 70% ice-cold acetone. Then, six parts of *n*-butanol/hydrochloric acid (37%) (95:5% *v*/*v*) were included and the resultant mixtures were heated at 95 °C for around one hour. Then, the solution mixture was left to reach RT, quenched by adding ammonium iron sulfate (NH_4_Fe(SO_4_)_2_) 2% (*w*/*v*), and then heated for a further 2 h at 80 °C. The absorbance of the cooled mixture was eventually quantified at 550 nm using an LT4500 reader. Based on a catechin calibration curve (linear range: 10–100 μg/mL, y = 0024x − 0.020, R^2^ > 0.996), the total amount of condensed tannins was calculated. The findings are presented as μg of catechin equivalents per gram of dry fraction.

### 4.7. Total Monoterpenoid Content Assessment

A technique previously reported by Ghorai et al. [101] was modified to assess the total monoterpenoid content. Namely, 200 μL of each extract (previously dissolved in methanol; 1 mg/mL) was completely combined with 1.5 mL of chloroform before standing for 3 min. Then, 100 μL of concentrated sulfuric acid was carefully added (under continuous cooling) and the suspensions were kept protected from light for 2 h in an orbital shaker. The formed precipitant was isolated, air-dried, and then dissolved in 95% (*v*/*v*) methanol. With an LT4500 microplate reader, the absorbance was measured at 538 nm. Based on a linalool calibration curve (linear range: 0–60 μM, y = 0.0050x + 0.0035, R^2^ > 0.994), the total monoterpenoid content was calculated. The findings were given as μg of linalool equivalents per g of dry fraction.

### 4.8. Total Soluble Sugar Content (TSSC) Assessment

The procedure used to assess the TSSC was modified somewhat from what had previously been described [99]. In a nutshell, a mixture of equal volumes of each fraction (separately) and concentrated sulfuric acid was shaken for half an hour at RT. The resulting mixture was then heated at 90 °C for 5 min after 30 μL of 5% phenol was introduced to each fraction. An LT4500 microplate reader was used to quantify the absorbance of the cooled solutions at 490 nm. The mannose calibration curve, with a linear range of 0–100 nM and a y = 0.021x + 0.019 R^2^ > 0.999, was used to compute the TSSC. The findings were given as nmol of mannose equivalents per gram of dry fraction.

### 4.9. Total Soluble Protein Content (TSPC) Assessment

To calculate the TSPC, 50 mg of each dry fraction was taken up in distilled water and vortexed extensively for 50 min. The BCA kit was used to measure the amount of protein in the sample, and an LT4500 reader was employed to quantify the absorbance at 562 nm. A standard curve for BSA (linear range: 0–2 mg/mL, y = 0.69x + 0.14 R^2^ > 0.995) was used to determine the TSPC. The protein content was calculated as mg of protein per gram of dry fraction.

### 4.10. Pigments Assessment

The total contents of chlorophyll-a and -b, lycopene, and β-carotene were assessed as formerly reported [99]. The following formulas were applied to calculate each content (1)–(4).

(1) $$\mathrm{Chlorophyll}\text{-}a\;(\mu g/g\;\mathrm{of}\;\mathrm{dry}\;\mathrm{fraction})=\left[\frac{\left(0.999A_{663}-0.0989A_{645}\right)}{20}\right]$$

(2) $$\mathrm{Chlorophyll}\text{-}b\;(\mu g/g\;\mathrm{of}\;\mathrm{dry}\;\mathrm{fraction})=\left[\frac{\left(1.77A_{663}-0.328A_{645}\right)}{20}\right]$$

(3) $$\mathrm{Lycopene}\;(\mu g/g\;\mathrm{of}\;\mathrm{dry}\;\mathrm{fraction})=\left[\frac{\left(-0.0458A_{663}+0.204A_{645}+0.372A_{505}-0.0806A_{453}\right)}{20}\right]$$

(4) $$\beta \text{-}\mathrm{carotene}\;(\mu g/g\;\mathrm{of}\;\mathrm{dry}\;\mathrm{fraction})=\left[\frac{\left(0.216A_{663}-1.224A_{645}-0.304A_{505}+0.452A_{453}\right)}{20}\right]$$

The findings are provided as values of μg of pigment (chlorophyll-a or -b, lycopene, or *β*-carotene) per gram of dry fraction.

### 4.11. Preparation of Standards and Samples

The stock solutions of the standards were made in methanol, except luteolin-7-*O*-glucoside in an acetonitrile/water mixture (1:1); 2′-hydroxyflavanone, 5-methoxyflavanone in a methanol/acetonitrile mixture (1:1); and apigenin, forsythoside-A, and verbascoside, which were solubilized in aqueous methanol (70% *v*/*v*) at a 1000 ppm concentration. The working solutions were created by adding ice-cold methanol to the respective standard stock solutions. To reduce the auto-oxidation of polyphenols (mainly flavonoids), every solution was kept darkened and shielded from light. Additionally, before application, the stock, standard, and sample solutions were maintained at −20 °C. Prior to the UPLC-ESI-MS/MS investigation, all solutions were subjected to membrane filtration (0.22 μm cellulose nitrate).

### 4.12. UPLC and MS

#### 4.12.1. Liquid Chromatography (LC) Conditions

We employed a Waters Acquity UPLC system (Waters Corp., Milford, MA, USA), which included an autosampler chamber, two pumps, and a degasser. The chromatographic separation was carried out using an ACQUITY UPLC BEH C18 (100 × 2.1 mm, particle size: 1.7 μm) column, heated to 30 °C, and eluted as previously published by Zhu et al. (2020), with some modifications [102]. In a nutshell, the mobile phase was composed of acetonitrile (eluent A) and formic acid 0.1% (*v*/*v*) (eluent B) solution. With a flowrate of 0.3 mL/min, linear gradient conditions of 5–100% A (0–4 min), 100–90% A (4.0–4.1 min), 90%A (4.1–5 min), 90–5% A (5–5.01 min), and 5% A (5.1–6 min) were applied. The autosampler was set to 4 °C and the injection volume was 10 μL.

#### 4.12.2. MS/MS Conditions

A Xevo Triple Quatrable mass spectrometer detector (Waters Corp.) was employed in the MS/MS studies. It was run in either positive or negative ionization mode. The selected MRM mode was employed to perform the quantitative study. Prior to the sample analysis, each standard underwent MS manual tuning to optimize the MRM conditions at a concentration of 1 ppm (Table 1 and Table 2, Figure 1). The following optimum tuning parameters were used to obtain the highest signal levels: 3.0 kV; cone voltage: 36 V; source temperature: 150 °C; disolvation temperature: 500 °C; source disolvating gas flow: 1000 L/h and gas flow: 20 L/h. High-purity nitrogen gas was utilized as the drying and nebulizing gas, while ultra-high-purity argon was employed as a collision gas. MassLynx software was employed for data collection and processing (version 4.1, Waters Co., Milford, MA, USA).

#### 4.12.3. Standardization of UPLC and MS Conditions

To optimize the experimental setting for the separation of the analytes and the production of symmetrical signal peaks, a thorough adjustment of the key parameters pertaining to the mobile phase, elution mode, flow rate, and stationary phase was carried out. Solvents such as acetonitrile/water and methanol/water in a variety of ratios were explored to determine the best mobile phase combination, but none of them produced symmetrical peaks or allowed for efficient separation. The water was made acidic for this purpose by adding formic acid at a concentration of 0.1% (*v*/*v*). The ionization of the analytes was assisted by the substitution of water with formic acid, which also promoted the peak symmetry and shape and enhanced the extract separation. The best separation with regard to the stationary phase was accomplished on an ethylene-bridged hybrid (BHE) column heated to 40 °C. The ESI was used in either negative or positive (ESI±) mode for the fragmentation of polyphenolic substances.

#### 4.12.4. Method Validation

The International Conference of Harmonization standards were followed [48]. For each of the studied analytes, the parameters linearity, limits of detection (LOD) and quantification (LOQ), precision, and accuracy were identified and assessed (Table S2). The generated standard curves of the standards were plotted using a linear regression equation of response peak areas as a function of various concentrations of compounds ranging from 0 to 500 ppb. All examined substances demonstrated good linearity, while their correlation coefficients (R2) were >0.99 (Table S2). Lastly, the reproducibility of the analytical methodology by means of the percentage of recovery (Table S2) was assessed. In this connection, each fraction of *S. scardica* was spiked with each standard solution of polyphenolic compound. The findings of at least six repetitions were obtained from spike samples that were generated in triplicate. According to the mathematical relation (5), where A is the final quantity detected, A0 is the initial amount, and Aa is the added amount, the percentage of recovery was calculated as follows:

% recovery = ((A − A0)/Aa) × 100% (5)

The precision and reproducibility of the aforementioned methodological approach were demonstrated by the average recovery rates of all polyphenolic compounds detected, which varied from 86.3% to 102.6%.

#### 4.12.5. Linearity, Accuracy, and Precision of the Methodology

The LOD and LOQ values, which were computed using the signal-to-noise (S/N) ratios, set at 3 and 10, respectively, were used to assess the specificity and selectivity of the analytical process. For polyphenolic substances, the LOD and LOQ ranges were 0.56–109.9 ppb and 1.11–105.2 ppb, respectively. With regard to the values in Table S2, of all the polyphenolic compounds listed, forsythoside-A had the lowest LOD and gallic acid had the highest, indicating that forsythoside’s sensitivity to detection was significantly higher than that of the other polyphenolic compounds that had been ionized under ESI. In contrast, luteolin-7-*O*-glucoside and 7-hydroxyflavanone were shown to have the maximum sensitivity when ionized in ESI+ mode compared to the other compounds. By computing the percentage of relative standard deviation (% RSD), it was then possible to assess how comparable the different samples were to one another within the same uniform. Six replicated samples of equal concentrations were examined within one day for their intraday accuracy and within six consecutive days for their interday accuracy to calculate the % RSD. In particular, the intra- and interday RSD findings for the polyphenolic chemicals ranged from 0.38 to 4.15% (Table S2).

### 4.13. DPPH Free Radical Scavenging Assay

The antioxidant activity was assessed using the DPPH^•^ assay in accordance with the approach reported by Parejo et al., with slight changes, with the use of the stable radical 1,1-diphenyl,2- picrylhydrazyl (DPPH^•^) [51,103]. To assess the antioxidant potential of all eight fractions, several extract concentrations were used. Briefly, 25 μL aliquots of the diluted fractions were dissolved in 975 μL of the mother DPPH^•^ solution (2 × 10^−5^ M in methanol), respectively, then the mixtures were vortexed and the reaction mixtures were left at RT. The absorbance was recorded at 517 nm at various time intervals using a UV–Vis spectrophotometer. Finally, a reduction in absorbance was determined when the reaction reached the maximum. The absorbance of the fractions in the absence of DPPH^•^ was subtracted from the corresponding absorbance with DPPH^•^. The calibration curve was used to compute the concentration of DPPH^•^ in the medium. The proportion of DPPH^•^ still present in the steady state for each fraction concentration examined was determined using the formula below: percentage of remaining DPPH^•^ = [DPPH^•^]at _t=T_/[DPPH^•^]_at t=0_, where T is the time required to attain a steady state. The quantity of extract required to reduce the initial DPPH concentration by half was utilized to express the antioxidant capacity of each fraction (EC_50_). The formula for calculating the antiradical efficiency (AE) is AE = 1/EC_50_. Trolox was employed as a positive control.

### 4.14. Ferric-Reducing Antioxidant Power (FRAP) Assay

The FRAP assay was carried out using the approach used by Benzie et al. [104]. First, 200 mL properly diluted aliquots of each plant extract were mixed with 1800 mL of FRAP solution. The FRAP solution was composed of 37.5 mL of 0.3 M acetate buffer (pH 3.6), 3.75 mL of 10 mM TPTZ in 40 mM hydrochloric acid, and 3.75 mL of 20 mM iron(III) chloride solution. The mixtures were kept for 10 min at RT and the absorbance was quantified at 593 nm with a V-630 Jasco UV–Vis spectrophotometer. Using ascorbic acid and Trolox standard curves, the antioxidant capacity was quantified as ascorbic acid equivalents (AAE mol/g sample) and Trolox equivalent antioxidant capacity (TEAC mol/g sample).

### 4.15. Cell Culture

SH-SY5Y human neuroblastoma cells were supplied by ATCC (Manassas, VA, USA) and were grown in Dulbecco’s modified Eagle’s medium complemented with 10% fetal bovine serum, 5% horse serum, 2 mM glutamine, and 1% antibiotics (penicillin and streptomycin). The cells were kept at 37 °C in a cell culture incubator under 5% CO_2_. The SH-SY5Y cells were differentiated after supplementing their medium with 10 μM retinoic acid for one week.

### 4.16. Dichlorofuoresence Diacetate (DCF-DA) Assay

The DCF-DA assay, in which DCF-DA is oxidized by reactive oxygen species (ROS) to its fluorescent form dichlorofluorescein (DCF), was used to identify the presence of intracellular ROS [105]. Here, 15,000 SH-SY5Y cells per well in a black clear-bottomed 96-well plate (SPL Life Sciences, Naechon-myeon, South Korea) were plated overnight, and the next day they were treated with 20 μM of DCF-DA solution for 45 min in a humidified incubator. Next, the cells were incubated with various SS fraction concentrations and 50 μM of hydrogen peroxide for 1 h. The fluorescence intensities of each well were quantified at 535 nm in a Synergy H1 microplate reader (Biotek, VT, USA) using a 485 nm excitation wavelength. Trolox was used as a standard antioxidant. Five independent experiments were performed. The EC_50_ values were estimated online with the use of an EC_50_ calculator made by AAT Bioquest [106].

### 4.17. Peptides Preparation

After the Aβ_25–35_ peptides were diluted in distilled water at a 1 mM concentration, aggregation formation was enabled by incubating the solution at 37 °C for a week. Before usage, the peptides were kept in aliquots and frozen at −20 °C.

### 4.18. MTT Assay

The MTT colorimetric assay was conducted to investigate the SH-SY5Y cell viability after treatment with SS plant extracts, or Aβ_25–35_ peptides, or the combination of both extracts and peptides [107]. First, 30,000 cells were placed in each well in clear 96-well plates. The following day, the cells were treated with different concentrations of the fractions, or 30 μM of Aβ_25–35_ peptides, or the combination of both for two days. Then, the cells were treated with 45 μg/mL of thiazolyl blue tetrazolium bromide at 37 °C, and after 4 h the media was aspirated and 150 μL of DMSO was poured into every well to solubilize the dark blue formazan crystals that were formed. The plate was agitated for 30 min on an orbital shaker with foil covering it. Using a Synergy H1 reader, the absorbance was quantified at 570 nm. After subtracting the blank reading, the absorbance of the treated cells was determined as a percentage in relation to the control. Five separate experiments were performed. The EC_50_ values were estimated online with the use of an EC_50_ calculator made by AAT Bioquest [106].

### 4.19. Statistical Analysis

The reported data are averages of n replicates with SD or SEM values. Using the statistical software GraphPad Prism (version 9.3.1, GraphPad Software, San Diego, CA, USA), a one-way analysis of variance followed by Dunnett’s test was employed to evaluate the statistical importance of the variances between control and treated cells. Here, *p* < 0.05 was required for statistical significance.

## 5. Conclusions

Conclusively, we have demonstrated for the first time that SS extracts ameliorate Aβ_25–35_ toxicity. Together with their antioxidant capacity, SS extracts may be useful for developing herbal drugs and functional food products or supplements that may alleviate AD.

## Figures and Tables

**Table 1:** Quantitative information illustrating the phytochemical contents of the four isolated (petroleum—SSPE; dichloromethane—SSDM; methanolic—SSM; initial aqueous—SSW1) extracts of *S. scardica.* The results represent the means ± SD of six separate studies. Tukey’s test (*p* < 0.05) showed that means and standard deviations (SDs) proceeded by different letters in the same row importantly vary from one another.

Chemical Class/ Analyte	Extracts of *Sideritis scardica*				
	SSPE	SSDM	SSM	SSW1	Expression Units
**Phenolics**					
Hydroxybenzoic acids					
4-hydroxybenzoic acid	*-*	0.34 ± 0.02 ^a^	32.16 ± 6.16 ^b^	150.42 ± 8.21 ^c^	μg/g of dry fraction
Protocatechuic acid	*-*	1.21 ± 0.03 ^a^	62.11 ± 4.59 ^b^	148.66 ± 11.36 ^c^	
Gallic acid	*-*	0.24 ± 0.16 ^a^	68.21 ± 4.00 ^b^	89.54 ± 2.36 ^c^	
Vanillic acid	0.22 ± 0.01 ^a^	3.21 ± 0.99 ^b^	32.70 ± 2.25 ^c^	67.65 ± 2.36 ^d^	
Syringic acid	*-*	*-*	73.42 ± 5.32 ^a^	235.69 ± 11.21 ^b^	
Hydroxycinnamic acids					
*p*-coumaric acid	*-*	1.31 ± 0.01 ^a^	93.21 ±1.05 ^c^	13.35 ± 1.49 ^b^	μg/g of dry fraction
Caffeic acid	0.15 ± 0.01 ^a^	1.21 ± 0.02 ^b^	78.99 ±3.42 ^c^	76.87 ± 8.99 ^c^	
Ferulic acid	1.23 ± 0.08 ^a^	1.12 ± 0.79 ^a^	55.65 ±4.42 ^b^	179.54 ± 6.35 ^c^	
Rosmarinic acid	*-*	1.11 ± 0.04 ^a^	0.042 ± 0.01 ^b^	*-*	
Chlorogenic acid	0.56 ± 0.04 ^a^	11.96 ± 2.34 ^b^	1036.20 ± 215.36 ^c^	1987.48 ± 102.21 ^d^	
Forsythoside A	24.54 ^a^	198.27 ± 10.21 ^b^	25612.65 ± 542.03 ^c^	39564.00 ± 496.22 ^d^	
Verbascoside	69.54 ^a^	258.98 ± 1.99 ^b^	32148 ± 1949.45 ^c^	48768.16 ± 2020.97 ^d^	
Ellagitannins					
Ellagic acid	0.79 ± 0.05 ^a^	2.37 ± 0.01 ^b^	9.65 ± 1.21 ^c^	8.01 ± 1.87 ^c^	μg/g of dry fraction
**Total Phenolic Content**	23.11 ± 1.21 ^a^	452.36 ± 18.32 ^b^	697.47 ± 15.54 ^c^	814.56 ± 31.21 ^d^	μg of gallic acid eq./g of dry fraction
**F** **lavonoids**					
Flavanones					
2′-hydroxyflavanone	*-*	2.22 ± 0.17 ^a^	*-*	284.69 ± 15.61 ^b^	μg/g of dry fraction
7-hydroxyflavanone	*-*	*-*	*-*	170.20 ± 9.61	
4′-methoxyflavanone	6.46 ± 0.04	*-*	*-*	*-*	
5-methoxyflavanone	8.13 ± 0.82	6.21 ± 0.06	*-*	*-*	
Flavones					
Apigenin	0.89 ± 0.09 ^a^	1.54 ± 0.08 ^b^	119.37 ± 8.36 ^c^	197.86 ± 2.90 ^d^	μg/g of dry fraction
Apigenin-7-*O*-glucoside	*-*	0.96 ± 0.01 ^a^	71.65 ± 0.56 ^b^	133.42 ± 11.40 ^c^	
Luteolin-7-*O*-glucoside	*-*	*-*	75.49 ± 3.03 ^a^	176.24 ± 9.01 ^b^	
Isorhamnetin	1.00 ± 0.01 ^a^	5.92 ± 0.49 ^b^	14.23 ± 0.11 ^c^	10.63 ± 0.03 ^d^	
Flavanols					
Quercetin-3-*O*-rhamnoside	*-*	2.42 ± 0.11 ^a^	94.56 ± 3.65 ^b^	182.51 ± 9.57 ^c^	μg/g of dry fraction
Quercetin-3-*O*-rutinoside	1.33 ± 0.13 ^a^	18.99 ± 2.27 ^b^	114.00 ± 13.21 ^c^	369.22 ± 20.39 ^d^	
Quercetin-3-*O*-galactoside	*-*	*-*	48.12 ± 3.21	53.61 ± 4.32	
Myricetin-3-*O*-galactoside	*-*	24.53 ± 2.11 ^a^	127.36 ± 10.37 ^b^	436.17 ± 18.15 ^c^	
Kaempferol-3-*O*-rutinoside	*-*	29.22 ± 2.48 ^a^	110.33 ± 9.65 ^b^	267.45 ± 18.12 ^c^	
Isoflavones					
Naringin	*-*	*-*	70.36 ± 1.47	*-*	μg/g of dry fraction
**Total Flavonoid Content**	148.59 ± 6.5 ^a^	223.51 ± 18.36 ^b^	897.24 ± 19.87 ^c^	1024.49 ± 36.14 ^d^	μg of rutin eq./g of dry fraction
	54.02 ± 3.64 ^a^	127.36 ± 9.63 ^b^	549.21 ± 21.98 ^c^	812.59 ± 28.36 ^d^	μg of catechin eq./g of dry fraction
**Total Condensed Tannins Content**	1.41 ± 0.02 ^a^	8.23 ± 0.23 ^b^	10.82 ± 1.01 ^c^	11.24 ± 0.83 ^c^	μg of catechin eq./g of dry fraction
**Total mono-Terpenoid Content**	3.27 ± 0.16 ^a^	26.23 ± 1.25 ^d^	9.46 ± 1.10 ^c^	7.26 ± 0.52 ^b^	μg of linalool eq./g of dry fraction
**Total Soluble Protein Content**	*-*	*-*	26.56 ± 2.36 ^a^	89.23 ± 3.16 ^b^	mg of BSA eq./g of dry fraction
**Total Soluble Sugar Content**	0.01 ± 0.00 ^a^	0.24 ± 0.02 ^b^	5.17 ± 0.28 ^c^	16.66 ± 0.37 ^d^	nmol of mannose eq./g of dry fraction
**P** **igments**					
Chlorophyll-a	26.74 ± 2.26 ^a^	69.54 ± 3.17 ^b^	149.36 ± 7.23 ^c^	298.15 ± 6.36 ^d^	μg of pigment/g of dry fraction
Chlorophyll-b	11.23 ± 0.14 ^a^	36.14 ± 1.32 ^b^	245.98 ± 14.11 ^c^	368.98 ± 15.14 ^d^	
β-Carotene	51.23 ± 0.01 ^c^	4.13 ± 0.28 ^b^	0.24 ± 0.36 ^a^	*-*	
Lycopene	79.13 ± 0.32 ^c^	10.56 ± 1.11 ^b^	1.42 ± 1.48 ^a^	*-*	

**Table 2:** Quantitative information illustrating the phytochemical contents of the four isolated (diethyl ether—SSDE; ethyl acetate—SSEA; butanol (organic phase)—SSB; butanol (aqueous phase)—SSW2) fractions of methanolic extract (SSM) of *S. Scardica.* The results represent the means ± SD of six separate studies. Tukey’s test (*p* < 0.05) showed that means and SDs proceeded by different letters in the same row importantly vary from one another.

Chemical Class/ Analyte	MeOH Fractions of *Sideritis scardica*				
	SSDE	SSEA	SSB	SSW2	Expression Units
**Phenolics**					
Hydroxybenzoic acids					
4-hydroxybenzoic acid	0.43 ± 0.02 ^a^	0.52 ± 0.13 ^a^	5.76 ± 0.001 ^c^	1.33 ± 0.02 ^b^	μg/g of dry fraction
Protocatechuic acid	*-*	0.40 ± 0.09 ^a^	6.52 ± 0.51 ^b^	32.56 ± 2.69 ^c^	
Vanillic acid	0.12 ± 0.06 ^a^	3.21 ± 0.23 ^b^	10.02 ± 1.00 ^c^	*-*	
Syringic acid	*-*	0.40 ± 0.17 ^a^	10.99 ± 0.69 ^c^	6.99 ± 0.42 ^b^	
Hydroxycinnamic acids					
*p*-coumaric acid	*-*	*-*	5.05 ± 0.12 ^b^	1.46 ± 0.00 ^a^	μg/g of dry fraction
Caffeic acid	0.38 ± 0.00 ^a^	0.44 ± 0.00 ^a^	12.69 ± 0.61 ^c^	7.57 ± 0.06 ^b^	
Ferulic acid	*-*	*-*	6.59 ± 0.21 ^b^	1.01 ± 0.00 ^a^	
Rosmarinic acid	*-*	*-*	6.32 ± 0.05	*-*	
Chlorogenic acid	0.40 ± 0.00 ^b^	0.07 ± 0.00 ^a^	6.12 ± 0.42 ^c^	36.21 ± 3.01 ^d^	
Forsythoside A	*-*	0.06 ± 0.00	*-*	*-*	
Verbascoside	*-*	*-*	7.55 ± 0.10 ^b^	1.27 ± 0.06 ^a^	
Ellagitannins					
Ellagic acid	4.00 ± 0.07 ^d^	0.02 ± 0.00 ^a^	2.01 ± 0.07 ^c^	0.30 ± 0.00 ^b^	μg/g of dry fraction
**Total Phenolic Content**	54.32 ± 3.67 ^a^	189.74 ± 4.26 ^b^	284.31 ± 16.32 ^c^	365.28 ± 16.83 ^d^	μg of gallic acid eq./g of dry fraction
**Flavonoids**					
Flavones					
Apigenin	5.00 ± 0.01 ^a^	*-*	*-*	30.11 ± 2.36 ^b^	μg/g of dry fraction
Apigenin-7-*O*-glucoside	*-*	*-*	7.62 ± 0.25	8.36 ± 1.37	
Luteolin-7-*O*-glucoside	5.99 ± 0.49 ^b^	0.05 ± 0.00 ^a^	8.45 ± 0.79 ^c^	12.34 ± 1.01 ^d^	
Flavanols					
Quercetin-3-*O*-rhamnoside	1.31 ± 0.01 ^b^	0.09 ± 0.00 ^a^	9.42 ± 0.66 ^c^	1.23 ± 0.04 ^b^	μg/g of dry fraction
Quercetin-3-*O*-rutinoside	*-*	*-*	*-*	1.54 ± 0.11	
Quercetin-3-*O*-galactoside	*-*	*-*	*-*	0.88 ± 0.00	
Myricetin-3-galactoside	4.62 ± 0.37 ^d^	1.08 ± 0.02 ^c^	0.30 ± 0.01 ^b^	0.05 ± 0.00 ^a^	
Kaempferol-3-*O*-rutinoside	*-*	*-*	2.31 ± 0.09 ^b^	1.11 ± 0.08 ^a^	
Isoflavones					
Naringin	*-*	0.06 ± 0.00 ^a^	6.12 ± 0.32 ^c^	1.51 ± 0.08 ^b^	μg/g of dry fraction
**Total Flavonoid Content**	184.33 ± 6.4 ^a^	289.34 ± 3.68 ^b^	423.66 ± 19.85 ^c^	547.59 ± 17.78 ^d^	μg of rutin eq./g of dry fraction
	86.34 ± 4.56 ^a^	453.97 ± 19.63 ^c^	697.74 ± 19.95 ^d^	190.16 ± 8.88 ^b^	μg of catechin eq./g of dry fraction
**Total Condensed Tannins Content**	4.05 ± 0.38 ^b^	*-*	4.33 ± 0.02 ^b^	1.23 ± 0.00 ^a^	μg of catechin eq./g of dry fraction
**Total mono-Terpenoid Content**	1.99 ± 0.00 ^b^	3.27 ± 0.00 ^d^	2.60 ± 0.07 ^c^	0.03 ± 0.00 ^a^	μg of linalool eq./g of dry fraction
**Total Soluble Protein Content**	*-*	*-*	0.04 ± 0.00 ^b^	0.026 ± 0.00 ^a^	mg of BSA eq./g of dry fraction
**Total Soluble Sugar Content**	*-*	*-*	*-*	0.04 ± 0.00	nmol of mannose eq./g of dry fraction
**Pigments**					
Chlorophyll-a	1.00 ± 0.03 ^a^	4.03 ± 0.78 ^b^	127.45 ± 6.32 ^c^	*-*	μg of pigment/g of dry fraction
Chlorophyll-b	12.25 ± 0.37 ^b^	0.24 ± 0.00 ^a^	100.99 ± 8.11 ^c^	9.23 ± 0.25 ^b^	
β-Carotene	*-*	*-*	*-*	*-*	
Lycopene	*-*	*-*	*-*	*-*	

**Table 3:** Antioxidant capacity levels of the different mixtures derived from SS aerial parts, as estimated by DPPH⋅, FRAP, and DCF-DA assays. Results are shown as average values ± standard deviations. EC_50_ in DPPH corresponds to the concentration of antioxidants necessary to decrease the initial DPPH by half and is measured in mg antioxidant/mg DPPH. EC_50_ in DCF-DA corresponds to the concentration of fraction required to decrease by 50% the presence of radical oxygen species, induced by 50 μM of H_2_O_2_.

		SSPE	SSDM	SSM	SSW1	SSDE	SSEA	SSB	SSW2
**DPPH**	**EC** **_50_**	-	13.38 ± 0.10	2.17 ± 0.06	1.86 ± 0.07	2.88 ± 0.30	0.44 ± 0.02	0.16 ± 0.02	5.40 ± 0.23
	**AE**	-	0.08	0.46	0.54	0.35	2.28	6.25	0.19
**FRAP**	**μ** **mol** **AAE/g**	49.5 ± 13.7	234.4 ± 31.2	963.9 ± 26.2	256.2 ± 16.0	585.7 ± 51.1	2263.5 ± 79.5	2707.6 ± 28.2	234.4 ± 2.1
	**μ** **mol** **TEAC/g**	60.3 ± 19.8	254.0 ± 35.6	1024.2 ± 60.7	275.9 ± 27.8	616.5 ± 34.5	2332.4 ± 68.7	2854.9 ± 71.0	252.1 ± 11.9
**DCF-DA assay**	**EC** **_50_** **μ** **g/mL**	-	2.69	2.47	40.86	0.62	0.57	2.06	12.65

**Table 4:** Cytotoxic potency of SS extracts in SH-SY5Y cells in terms of half-maximal effective concentrations. EC_50_ corresponds to the concentration of extract required to reduce the cell viability by 50%.

		SSPE	SSDM	SSM	SSW1	SSDE	SSEA	SSB	SSW2
**Cell viability assay**	**EC_50_** **(μg/mL)**	249.09	477.25	451.85	534.18	362.39	470.65	631.59	601.71

## Data Availability

The data are contained within the article.

## Supplementary Materials

The following supporting information can be downloaded at: https://www.mdpi.com/article/10.3390/plants12081716/s1, Table S1: Multiple Reaction Monitoring conditions for polyphenolic acids and flavonoids in UPLC-MS/MS analysis; Table S2: The limit of detection (LOD), quantification (LOQ), linearity, precision, and accuracy results for the screened polyphenolic compounds.
