# Supplementary material for: Sideritis scardica Extracts Demonstrate Neuroprotective Activity against Aβ25–35 Toxicity
Source: Plants (Basel). 2023 Apr 20;12(8):1716. doi: 10.3390/plants12081716 (PMC10142657; doi:10.3390/plants12081716)
Supplement: Supplementary file 1 [file plants-12-01716-s001.zip › plants-2317235-supplementary.pdf]

Sideritis scardica extracts demonstrate neuroprotective activity against Aβ_{25-35} toxicity

Antonis Ververis [1], Kristia Ioannou [1], Sotiris Kyriakou [2], Niki Violaki [3], Mihalis I. Panayiotidis [2], Michael Plioukas [3] and Kyproula Christodoulou [1,*]

[1] Neurogenetics Department, The Cyprus Institute of Neurology and Genetics, Nicosia 2371, Cyprus
[2] Department of Cancer Genetics, Therapeutics & Ultrastructural Pathology, The Cyprus Institute of Neurology & Genetics, Nicosia 2371, Cyprus
[3] Department of Life and Health Sciences, School of Sciences and Engineering, University of Nicosia, Nicosia 2417, Cyprus
* Correspondence: roula@cing.ac.cy; Tel.: +357-22-392649

Supplementary Material

Table S1: Multiple Reaction Monitoring conditions for polyphenolic acids and flavonoids in UPLC-MS/MS analysis

Polyphenolic compound	Chemical formula	Molecular weight	$[M-H]^{\pm}$ (m/z)	MS^2 fragments (m/z)	Cone voltage (V)	Collision energy (eV)	Retention time (R_t)
4-hydroxybenzoic acid	$C_7H_6O_3$	138.12	136.95	65.0<br>93.0	23	25<br>13	1.88
Protocatechuic acid	$C_7H_6O_4$	154.12	152.95	108.95	25	13	1.64
Gallic acid	$C_7H_6O_5$	170.12	168.95	78.98<br>124.95	23	22<br>15	1.37
Vanillin	$C_8H_8O_3$	152.15	151.0	92.2<br>136.0	22	20<br>15	2.23
Syringic acid	$C_9H_{10}O_5$	198.17	197.0	122.95<br>182.0	27	23<br>13	1.93
p-coumaric acid	$C_9H_8O_3$	164.16	163.0	119.0	15	13	2.13
Caffeic acid	$C_9H_8O_4$	180.16	178.95	134.95	25	13	1.89
Ferulic acid	$C_{10}H_{10}O_4$	194.18	192.95	134.0<br>178.0	26	25<br>12	2.20
Rosmarinic acid	$C_{18}H_{16}O$	360.32	359.2	161.0<br>197.0	10	15<br>15	2.26
Chlorogenic acid	$C_{16}H_{18}O_9$	354.31	353.1	84.0<br>191.02	22	14<br>14	1.70
Forsythoside A	$C_{29}H_{36}O_{15}$	624.59	623.65	160.90	35	27	4.48
Verbascoside	$C_{29}H_{36}O_{15}$	624.59	623.00	191.0	35	28	3.29
Ellagic acid	$C_{14}H_6O_8$	302.19	301	145.0<br>173.0	35	34<br>36	2.00
2′-hydroxyflavanone	$C_{15}H_{12}O_3$	240.27	239	119.3<br>93.1	40	25<br>16	3.42
7-hydroxyflavanone	$C_{15}H_{12}O_3$	240.27	239.05	135.2<br>91.15	41	25<br>23	3.52
4′-methoxyflavanone	$C_{16}H_{14}O_3$	254.29	255.15	240<br>161.3	31	17<br>22	3.78
5-methoxyflavanone	$C_{16}H_{14}O_3$	254.29	255.15	151.3	34	22	3.49
Apigenin	$C_{15}H_{10}O_5$	270.05	269.95	125	29	23	2.35
Apigenin-7-*O*-glucoside	$C_{21}H_{20}O_{10}$	432.38	431.15	268.35	35	22	2.15
Luteolin-7-*O*-glucoside	$C_{21}H_{20}O_{11}$	448.38	449.15	287.1	34	31	2.01

Isorhamnetin	$C_{16}H_{12}O_7$	316.28	315	151.0<br>300.2	43	30<br>20	2.86
Quercetin-3-*O*-rhamnoside	$C_{21}H_{20}O_{11}$	448.38	447.01	271.0<br>300.0	43	47<br>28	2.14
Quercetin-3-*O*-rutinoside	$C_{27}H_{30}O_{16}$	610.53	609.1	300.0<br>271	47	39<br>65	1.92
Quercetin-3-*O*-galactoside	$C_{21}H_{20}O_{12}$	464.38	463.3	300.0<br>271.15	47	24<br>44	1.99
Myricetin-3-*O*-galactoside	$C_{21}H_{20}O_{13}$	480.38	479.05	271.1<br>287.1	48	39<br>44	1.87
Kaempferol-3-*O*-rhamnoside	$C_{21}H_{20}O_{10}$	432.39	431.05	255.3<br>284.2	45	42<br>28	2.27

Table S2: The limit of detection (LOD), quantification (LOQ), linearity, precision, and accuracy results for the screened polyphenolic compounds. The calibration equations represent the peak area as a function of concentration in ppb. The intra- and interday experimental data concern data that have been collected from a six days experiment, whereas the %recovery data are the means of three independent experiments.

Compound	Linear range (ppb)	LOD (ppb)	LOQ (ppb)	Calibration equation[a]	Correlation coefficient (r^2)	%RSD (intra-day)[b]	%RSD (inter-day)[c]	%REC[d]
POLYPHENOLIC COMPOUNDS								
4-hydroxybenzoic acid	3.01-499.50	3.01	14.20	y=36.87x-62.07	0.9991	1.15	2.21	98.8
Protocatechuic acid	0.66-504.50	0.66	14.70	y=34.24x-69.4	0.9995	1.25	2.65	86.3
Gallic acid	53.20-513.20	53.20	105.20	y=0.67x-1.5	0.9996	0.46	0.21	99.9
Vanillin	2.87-335.00	2.87	5.62	y=0.67x-0.1	0.9999	0.98	0.95	100.4
Syringic acid	2.01-501.60	2.01	2.86	y=7.28x-2.7	0.9996	1.36	1.01	96.6
p-coumaric acid	0.65-497.30	0.65	1.55	y=52.84x+36.9	0.9997	1.7	1.94	93.2
Caffeic acid	1.21-500	1.21	1.25	y=92.95x-344.4	0.9995	1.01	2.21	100.1
Ferulic acid	2.10-505.60	2.10	12. 17	y=19.02x-68.4	0.9992	0.7	2.45	102.6
Rosmarinic acid	2.32-499.50	2.32	2.56	y=7.03x+12.34	0.9996	1.3	3.02	86.9
Chlorogenic acid	3.48-495.60	3.48	4.76	y=25.02x+60.3	0.9991	1.35	1.98	87.4
Forsythoside-A	0.56-499.99	0.56	4.15	y=26.08x-1.36	0.9995	1.11	2.00	89.9
Verbascoside	0.62-486.86	0.62	11.62	y=16.789x-62.21	0.9999	1.06	4.15	94.3
Ellagic acid	5.53-499.10	5.53	75.60	y=2.18x+7.4	0.9995	1.32	3.05	89.9
2′-hydroxyflavanone	19.50-250.00	19.50	20.12	y=38.69x+22.5	0.9998	2.7	4.32	99.5
7-hydroxyflavanone	1.97-249.90	1.97	2.21	y=51.17x-73.6	1	2.63	1.42	98.9
4′-methoxyflavanone	2.21-250.00	2.21	3.89	y=83.54x+60.3	0.9999	2.89	1.87	93.6
5-methoxyflavanone	6.47-248.50	6.47	8.52	y=195.14x-493.9	0.9992	3.21	2.69	94.7
Apigenin	0.59-249.99	0.59	7.98	y=45.12x+3.21	0.9994	0.38	1.87	96.6
Apigenin-7-*O*-glucoside	1.87-125.30	1.87	4.42	y=6.17x+3.8	0.9998	3.48	2.54	95.8
Luteolin-7-*O*-glucoside	2.21-250.10	2.21	2.22	y=51.52x-89.9	0.9998	3.64	3.22	89.2

Isorhamnetin	14.01-251.1	14.01	2.31	y=6.08x-15.4	0.9992	2.48	1.18	100.1
Quercetin-3-*O*-rhamnoside	1.02-250.60	1.02	4.21	y=60.83x-38.6	0.9999	2.21	3.01	99.8
Quercetin-3-O-rutinoside	1.40-251.30	1.40	4.32	y=97.74x+109.7	0.9999	1.35	1.89	87.4
Quercetin-3-O-galactoside	6.32-249.90	6.32	3.21	y=3.97x+0.5	0.9998	2.14	1.37	96.3
Myricetin-3-galactoside	0.85-251.20	0.85	2.12	y=26.38x-31.8	0.9997	1.78	1.65	100.2
Kaempferol-3-*O*-rutinoside	0.76-250.00	0.76	1.21	y=25.73x+73.7	0.9997	1.36	2.21	91.2
Naringin	3.01-250.60	3.01	1.21	y=22.88x-43.3	0.9997	2.22	4.02	95.4

[a] Chromatographic peak area (y) as a function of ppb concentration (x)

[b] Values are means of intra-day assays (*n*=6)

[c] Values are means of inter-day assays (*n*=6)

[d] (*n*=3)
